# Macrophage IL-1β contributes to tumorigenesis through paracrine AIM2 inflammasome activation in the tumor microenvironment

**DOI:** 10.3389/fimmu.2023.1211730

**Published:** 2023-06-28

**Authors:** Zhi Huan Chew, Jianzhou Cui, Karishma Sachaphibulkij, Isabelle Tan, Shreya Kar, Kai Kiat Koh, Kritika Singh, Hong Meng Lim, Soo Chin Lee, Alan Prem Kumar, Stephan Gasser, Lina H. K. Lim

**Affiliations:** ^1^ Department of Physiology, Yong Loo Lin School of Medicine, National University of Singapore, Singapore, Singapore; ^2^ Immunology Translational Research Program, Yong Loo Lin School of Medicine, National University of Singapore, Singapore, Singapore; ^3^ Immunology Program, Life Sciences Institute, National University of Singapore, Singapore, Singapore; ^4^ Graduate School for Integrative Sciences and Engineering, National University of Singapore, Singapore, Singapore; ^5^ Cancer Science Institute of Singapore, National University of Singapore, Singapore, Singapore; ^6^ Department of Pharmacology, Yong Loo Lin School of Medicine, National University of Singapore, Singapore, Singapore; ^7^ Centre for Cancer Research, Yong Loo Lin School of Medicine, National University of Singapore, Singapore, Singapore; ^8^ Department of Haematology-Oncology, National University Hospital, Singapore, Singapore; ^9^ Department of Microbiology and Immunology, Yong Loo Lin School of Medicine, National University of Singapore, Singapore, Singapore; ^10^ Roche Pharma Research and Early Development, Roche Innovation Center, Roche Glycart AG, Schlieren, Switzerland

**Keywords:** innate immunity, inflammasome, cytosolic DNA, tumor microenvironment, AIM2 inflammasome

## Abstract

Intracellular recognition of self and non-self -nucleic acids can result in the initiation of effective pro-inflammatory and anti-tumorigenic responses. We hypothesized that macrophages can be activated by tumor-derived nucleic acids to induce inflammasome activation in the tumor microenvironment. We show that tumor conditioned media (CM) can induce IL-1β production, indicative of inflammasome activation in primed macrophages. This could be partially dependent on caspase 1/11, AIM2 and NLRP3. IL-1β enhances tumor cell proliferation, migration and invasion while coculture of tumor cells with macrophages enhances the proliferation of tumor cells, which is AIM2 and caspase 1/11 dependent. Furthermore, we have identified that DNA-RNA hybrids could be the nucleic acid form which activates AIM2 inflammasome at a higher sensitivity as compared to dsDNA. Taken together, the tumor-secretome stimulates an innate immune pathway in macrophages which promotes paracrine cancer growth and may be a key tumorigenic pathway in cancer. Broader understanding on the mechanisms of nucleic acid recognition and interaction with innate immune signaling pathway will help us to better appreciate its potential application in diagnostic and therapeutic benefit in cancer.

## Introduction

The innate immune system serves as the first-line defense against bacteria, viruses, and other pathogens(1). DNA is typically found in the nucleus of eukaryotic cells, and the presence of DNA in aberrant locations, such as the cytoplasm and endosomes triggers immune activation, sensed by a group of proteins collectively known as DNA sensors(2). We and others have reported that cytosolic DNA is found in cancer cells and tissues and not in healthy tissues, suggesting that it is a unique cancer phenotype (3). Whether the tumor cells or innate immune cells in the tumor microenvironment recognizes cancer derived cytosolic nucleic acids has been recently questioned, and the involvement of DNA sensors has appeared to be important both intrinsically and for tumor surveillance (4). Another class of DNA sensors are the inflammasomes and namely, the AIM2 inflammasome senses dsDNA(5) and has been implicated in the development (6) as well as regulation (7) of various cancers.

The inflammasomes are a family of oligomeric protein complex made up of the sensor component which, upon sensing of PAMPs and DAMPs, binds adaptor protein ASC, which activates caspase-1, and triggers the release of pro-inflammatory cytokines such as IL-1β, making the inflammasomes one of the critical drivers of inflammation(8). Inflammasomes are defined by their sensor, which include AIM2, Pyrin, and NLRP1 and NLRP3. The diversity and specificity of sensors allow responsiveness to a broad range of stimuli, either extrinsic (microbial molecules) or intrinsic (danger signals). Activation of inflammasomes requires two different signals, encompassing a priming step which usually involves microbial molecules which would induce nuclear factor kappa B (NF-κB)-dependent expression of the sensor protein and pro-IL-1β and a second signal provided by various structurally unrelated microbial molecules such as toxins or danger signals (e.g., DNA, ATP or uric acid), which, together with the first signal, will trigger inflammasome multimerization and activation(9). The AIM2 inflammasome consists of AIM2, an adaptor molecule ASC (apoptosis-associated speck-like protein containing a CARD), and pro-caspase-1. Upon inflammasome activation, caspase-1 is cleaved, which in turn, cleaves IL-1β and gasdermin-D which frees the N-terminal effector domain from the C-terminal inhibitory domain. Oligomerization of the gasdermin-D N terminal subsequently forms a pore in the membrane and leads to membrane rupture and pyroptosis.

As mentioned, AIM2 is a cytosolic DNA sensor protein which can have inflammasome dependent and independent functions. AIM2 has been implicated in the development of various cancers including prostate cancer, squamous cell carcinoma (10, 11), as well as non-small cell lung cancer through the regulation of mitochondrial dynamics (12) and renal cell carcinoma through FOXO3a-ACSL4 inhibition of ferroptosis(13). However, other studies have reported that AIM2 acts as a tumor suppressor gene in many cancers including colon cancer and endometrial cancer (6, 14),inhibiting cancer proliferation, invasion and migration through various mechanisms, including suppressing glioma-associated oncogene-1 (GLi1) (15), inactivating PI3K/AKT/mTOR signaling (16), Moreover, the trigger mechanisms of the AIM2 inflammasome remain unknown. Cytosolic DNA, including ssDNA, dsDNA, and DNA/RNA hybrids, are present in all tested cancer cell lines and cancer samples (3, 17). Strikingly, cytosolic DNA is not found in healthy tissues, suggesting that it is a unique cancer phenotype and hence giving us a clue to study the link between cancer cells and AIM2 inflammasome activation.

Based on previous reports that cancer cells contain cytosolic nucleic acids (3) and cancer cells can activate macrophages, we hypothesized that macrophages can be activated by tumor-derived nucleic acids to induce inflammasome activation in the tumor microenvironment, which in turn, can affect cancer progression.

## Materials and methods

### Mice

All animal work was approved by the National University of Singapore Institutional Animal Care and Use Committee (IACUC), an AALAAC accredited institution and was in accordance with the National Advisory Committee for laboratory Animal Research (NACLAR) Guidelines. TRAMP-C2 cells were injected subcutaneously into C57BL6 mice or AIM2-/- mice on the same background. Tumors were homogenized or treated with collagenase before isolating CD11b+ cells through a column and incubating overnight, after which supernatants were collected for ELISA analysis.

### Cell lines, drugs and treatments

Human THP-1, U937, HeLa cells, A549, HCT-116, and mouse TRAMP-C2 cells were purchased from ATCC (USA). A549, HeLa and TRAMP-C2 cells were grown in DMEM medium (Invitrogen, USA), while HCT116 cells were cultured in McCoy’s 5A medium (Invitrogen, USA), and both mediums are supplemented with 10% heat-inactivated FCS (Hyclone, USA) and 1% pen/strep (Invitrogen). THP-1 and U937 cells were cultured in RPMI medium (Hyclone, USA) supplemented with 10% heat-inactivated FCS (Hyclone, USA). All cells were grown at 37°C in a humidified 5% CO2 incubator (Thermo Scientific, USA). THP1 cells were differerentiated in PMA (#tlrl-pma, InvivoGen, USA) and stimulated with 1 μg/ml LPS (#tlrl-peklps, InvivoGen, USA) overnight before fresh cell medium was replaced and the cells were either treated with 50% cancer CM or transfected with 1 μg/ml poly (dA:dT) (#tlrl-patc, InvivoGen, USA) with lyovec transfection reagent. *Inhibitors* THP1 Cells were primed with 100 ng/ml PMA, followed by LPS (1 μg/ml) for 3 hours prior to treatment 1 hour with 1 μg/ml Poly(dA:dT) complexes (AIM2 inflammasome inducer) or A549 tumor CM in the presence of ODN TTAGGG (A151; AIM2 inhibitor, 0.5 μM) or MCC950 (NLRP3 inhibitor, 0.5 μM). Cell supernatants were detected using ELISA.

### Bone marrow derived macrophage isolation and differentiation

Bone marrow derived macrophages (BMDMs) from mice were obtained by flushing the femurs and tibias of mice with DMEM media. Briefly, red blood cells were removed through osmotic lysis and the bone marrow cell suspension was washed twice with PBS and cultured with BMDM media (DMEM medium containing 10% FBS (v/v), 100 U/mL penicillin, 100 µg/mL streptomycin, 2 mM L-glutamine and 20% L-929-conditioned DMEM (v/v) as a source of M-CSF). After 3 days of culture, the cells were supplemented with fresh BMDM media. At day 7, culture media and non-adherent cells were removed, and the remaining adherent cells were replenished with fresh BMDM media before experiments.

### Tumor conditioned media preparation

A549, Hela, MCF7 cells are cultured in T-75 flasks until 80% confluency before the existing media was replaced by new DMEM medium. The cells were grown for another 24 h in the new DMEM media before the conditioned media was collected and filtered using 0.45 µm filter (Millipore, USA) prior to use.

### Flow cytometry

Cells were stained with Fixable Viability Dye eFluor 506 (1:1000) in 1x PBS for 30 min at 4°C (Thermo Fisher Scientific, USA) before incubated with Fc block (BioLegend, USA) for 15 min at 4°C. Cells were stained with fluorescence-conjugated antibodies (BioLegend, USA) diluted in FACS buffer and incubated on ice for 30 min in the dark. Macrophages were identified as CD45+ Ly6G- Gr1- F4/80+ CD11b+ cells. Cells were analyzed on a BD LSRFortessa (BD Biosciences, USA) or Attune NxT Flow Cytometer (Thermo Fisher Scientific, USA). Data analysis was performed using FlowJo software (FlowJo, USA).

### Immunohistochemistry

Immunohistochemistry (IHC) was performed using the Leica Bond max autostainer (Leica Biosystems, Nussloch GmbH) and bond polymer refine detection kit (DS9800, Leica Biosystems) following the manufacturer’s instructions. Briefly, tissues were fixed in formalin, dehydrated and cleared before adding paraffin (pre-heated to 60oC) and left overnight. Sectioning was performed with a microtome. Formalin-fixed tissue sections were de-waxed, rehydrated and antigen retrieval was achieved using citrate buffer at pH 6 for 20 minutes. Endogenous peroxidase activity was quenched in 0.3% hydrogen peroxide for 10 minutes and non-specific sites were blocked with 3% bovine serum albumin for 20 minutes. Further, single immunostaining with primary antibodies for CD68 (1:50, Bio-Rad), inducible nitric oxide synthase (iNOS, 1:50, Abcam), mannose receptor CD206 (1:200, Abcam) followed by incubation with respective HRP-linked secondary antibodies (for CD68: goat anti-rat antibody Bio-Rad; for iNOS and CD206: goat anti-rabbit antibody, DAKO) was performed. 3,3’-diaminobenzidine (DAB) was used as the chromogen to obtain the brown colour. Counterstaining was with hematoxylin for 10 min. The mean score of five high-power fields (HPF) (×400) was counted for each slide under a light microscope (Olympus BX43) and digital images were acquired using Olympus cellSens Entry imaging software. In the IHC stained sections, macrophages observed with their cytoplasm stained tan or brown were considered positive.

### Immunofluorescence

Cytosolic dsDNA and DNA-RNA staining were performed as described previously (16). Briefly, 50% formamide (VWR International, USA) diluted in PBS was added for 10 mins at room temperature, followed by incubation for 15 mins at 75°C to denature dsDNA. Cells were washed with PBS and incubated with blocking buffer (1% BSA (Sigma Aldrich), 2% goat serum (Hyclone) in PBS) for 1 h to prevent non-specific binding of antibodies. Cells were then stained with dsDNA (1:200, Sigma-Aldrich, USA) or S9.6 DNA-RNA hybrids (1:100, Kerafast, Boston, USA) antibodies overnight in 4°C. Cells were subsequently washed with PBST (0.1% Tween) thrice followed by anti-mouse IgG coupled with Cy3 (Millipore, Singapore) or anti-rabbit IgG coupled to Alexa Fluor 488 (Invitrogen, Singapore). Finally, DNA fluorophore DAPI (0.5 μg/ml in PBS, KPL Inc., USA) was added for 10 min. Slides were washed once in PBS before mounted with Da-/- fluorescent mounting medium (Da-/-, UK). Confocal images of staining were captured using a Zeiss Axio Imager Z1 fluorescent microscope equipped with AxioVision 4.8 software (Carl Zeiss MicroImaging, USA) or confocal TCS SP5 (Leica, Singapore). Images were analyzed using Photoshop CS4 (Adobe, USA) or ImageJ. Colocalisation of AIM2 with DNA or other proteins were quantified using Metamorph (Metamorph NX, version 8.12, Molecular Devices, USA).

### Western blot

Cells were lysed with RIPA (Radio-Immunoprecipitation Assay) buffer containing protease inhibitors (Calbiochem, USA). 30 μg of total protein was loaded onto a SDSPAGE gel and electroblotted onto Hybond ECL membrane (GE Healthcare, UK). The blots were stained with primary antibodies (AIM2 (1:1000, Cell Signaling Technology, USA), NLRP1 (1:200, Santa Cruz Biotechnology, USA), NLRP2 (1:1000, Proteintech, USA), NLRP3 (1:1000, Adipogen, USA), NAIP (1:1000, Adipogen, USA), ASC (1:1000, Cell Signaling Technology, USA), Caspase 1 (1:200, Santa Cruz Biotechnology, USA), IL1β (1:1000, R & D, USA), Tubulin (!;1000, Cell Signaling Technology, USA), Actin (1:1000, Cell Signaling Technology, USA) overnight at 4°C after blocked with 5% low fat milk (Bio-Rad, USA). The blots were washed with TBST and incubated with a secondary HRP-linked antibody (1:5000, Santa Cruz Biotechnology, USA). Blots were washed TBST before visualization using ECL reagents according to manufacturer’s instructions (Perkin Elmer, USA). Each protein bands’ intensities were normalized against its loading control band intensity for quantification using ImageJ. Briefly, an area of interest rectangle was created around each band and the integrated density was measured, background subtracted, and divided by its specific loading control band.

### ELISA assay

Cytokines in culture supernatant and tissue lysates were measured by ELISA using commercially available kits. Mouse IL-1β (88-7013, eBioscience, USA) and human IL-1β (88-7261-86, eBioscience, USA) were used in accordance with the manufacturer’s protocol. Absorbance was read with a Tecan Spark 10M microplate reader (Tecan, USA) at absorbance values as indicated in the manufacturer’s protocol.

### Isolation of CD11b+ tumor-associated macrophages

Tumors were mechanically dissociated and strained through a 40 µm nylon mesh before digestion into single cells with collagenase type II (0.5 mg mL−1), collagenase type IV (0.5 mg mL−1), hyaluronidase (10 U/mL) and DNase I (0.01 mg mL−1) for 2 h at 37 °C. The dissociated cells were collected, lysed by RBC lysis buffer before CD11b+ tumor associated macrophages were isolated out of these suspensions by the MACS technology according to the manufacturer’s instructions using 10 μl CD11b microbeads (130049601; Miltenyi, USA) per 1x10^7^ cells. The isolated CD11b+ cells were grown overnight in complete RPMI media overnight before the cells and cell supernatants were collected for analysis.

### siRNA transfection

AIM2-specific siRNA and scrambled siRNA (Thermo Fisher) were used according to manufacturer’s instructions. THP-1 cells were seeded at a concentration of 5x105/well in a 12 well plate (Greiner Bio-science) before silencing with a final concentration 10 nM of siRNA and grown overnight before treatment with or without 100ng/ml PMA, 1 μg/ml LPS, and 50% A549 tumor conditioned media for another 24 h.

### MTT cell proliferation assay

A549 cells were seeded in 24 well plates and treated with IL-1β (#29-8018-65, Thermo Scientific, USA) at the indicated concentrations for 72 h, after which cell viability was measured using an MTT Cell Proliferation and Cytotoxicity Assay Kit (Boster Bio), according to the manufacturer’s instructions. Absorbance was measured using a TECAN spectrophotometer (Tecan Trading AG, Switzerland) at 490 nm.

### Migration and invasion assay

A549 cells were seeded into cell migration chambers (SPL, Korea) of PET membrane with 8.0 μm pore size. IL-1β was added to the cells and the chambers were incubated for 24 h in a humidified chamber at 37°C, 5% CO2. For the invasion assay, inserts were coated with 10% Matrigel prior to cell seeding. For co-culture, the bottom chamber was seeded with mouse BMDM cells stimulated with LPS (1 µg/ml) one day prior to TRAMPC2 cell seeding before the chambers were incubated for 24 h. At the end of the incubation period, the inserts were washed using 1xPBS. Inserts were fixed with 100% methanol for 15 minutes before washing and air drying, followed by cell staining using 0.5% crystal violet (filtered) for 30 minutes. The upper membrane of the inserts was rinsed and cleaned to remove excess stain and non-migrated cells. Five microscopic fields of each insert were captured using Nikon Stereoscopic Microscope SMZ1500 and Digital Camera DXM1200F (Nikon, Melvile, NY, USA).

### Statistics

Unpaired Student’s T-Test or One-way/Two-way analysis of variance (ANOVA) with Tukey’s post-test was performed when appropriate. Results are presented as mean values ± standard error (SE).

A p-value of < 0.05 or less was deemed significant.

## Results

### Higher dsDNA and infiltration of CD68+ and CD206+ cells in the mouse mammary tumor microenvironment

**Figure 1:**
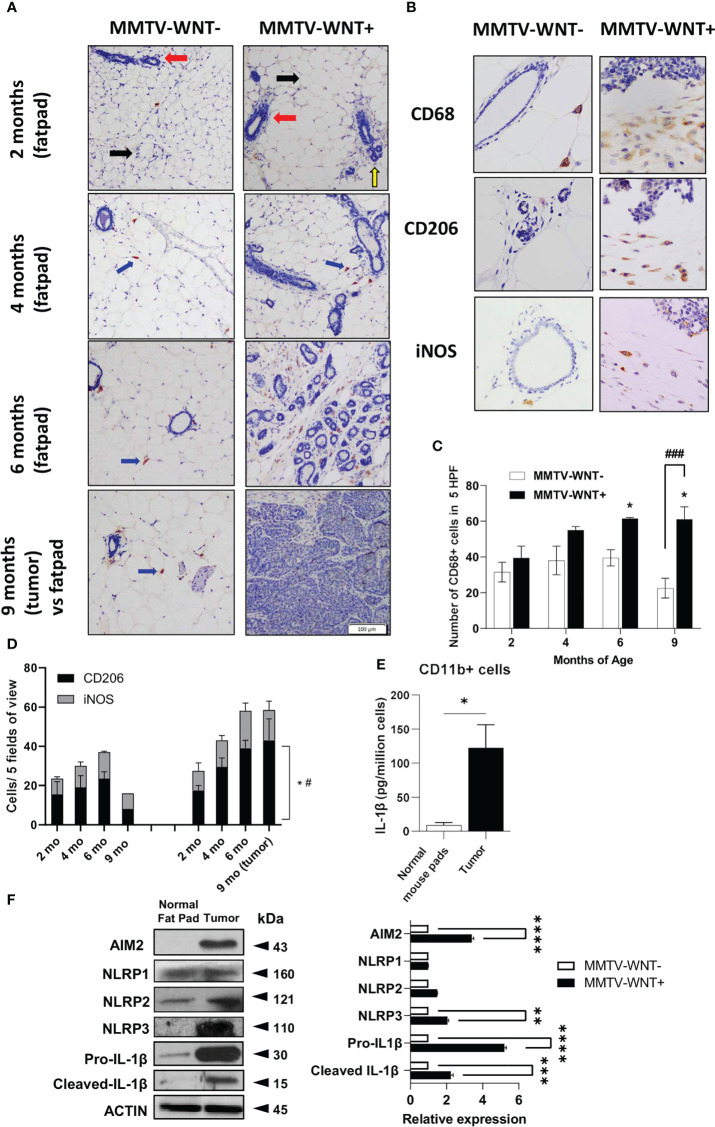
Higher dsDNA and infiltration of CD11b+ cells in the mouse mammary tumor microenvironment. **(A)** Immunohistochemical expression of CD68 and Haemotoxylin and Eosin staining of MMTV-WNT- and MMTV-WNT+ mammary fat pads at 2,4,6 and 9 months and upon tumor resection at 9 months. (Magnification, 100x). **(B)** Immunohistochemical expression of CD68 in 9-month MMTV-WNT- mammary fat pads and MMTV-WNT+ tumors. Blue corresponds to nuclear staining and brown corresponds to positive staining (magnification, 400x). **(C)** CD68+ cells were counted in 5 high power fields. **(D)** iNOS+ and CD206+ cells were counted in 5 high power fields. * P<0.05 vs 2 months; # P<0.05, ### P<0.001 vs MMTV-WNT- at same time point. 2 replicates per age were performed **(E)** CD11b+ macrophages were isolated from normal mouse pads and MMTV tumors and grown overnight. IL1β production was analyzed using ELISA. **(F)** Inflammasome components (IL1β, AIM2, NLRP1,2,3) and activation (cleaved) were analyzed using western blotting. Graph shows the quantification of immunoblots using Image J (fold change vs Actin) * P<0.05 from n=3-4 mice. **p<0.01; ***p<0.001 **** p<0.0001.

The presence of nucleic acids have been reported in cancer tissues and cell lines (3) which could activate DAMPs in tumor associated macrophages (TAMs). We first confirmed the expression of dsDNA and in a spontaneous breast cancer mouse model which develops tumors at ~6-9 months of age- MMTV-Wnt+ mice, compared to non-tumor fat pads. Indeed, tumors expressed more dsDNA compared to normal mammary tissue (**Figure S1A**). We next investigated the tumor microenvironment in MMTV-Wnt+ fatpads and tumor (which developed at 9 months) and MMTV-Wnt- fat pads. Many more CD11b+F480+ cells were present in tumor tissue (**Figure S1B**). Interestingly macrophage infiltration occurs early before tumor development, confirmed by immunohistochemistry of MMTV-Wnt+ and MMTV-Wnt- fat pads stained with CD68 and counterstained with Haemotoxylin (at 2,4,6 and 9 months of age). **Figure 1A** shows mouse mammary tissue composed of fat pads (black arrow), ducts (red arrow) and acinar structures (yellow arrow). MMTV-Wnt+ pads exhibit ductal and acinar proliferation with branching and budding, progressing from 2 months to 6 months in comparison to their respective age matched control littermates. Formation of malignant tumor is observed at 9 months which shows atypical epithelial cells forming enlarged irregular glands with intraluminal papillary projections. At places, there is also formation of cysts filled with secretory material and many solid areas of tumor cells can be seen. Macrophages were stained with CD68 (400x) and a significant increase in CD68+ cells was seen in fat pads from MMTV-WNT+ mice progressing from 2-6 months and tumors at 9 months (**Figures 1B, C**). In addition, staining of sections with an inflammatory macrophage marker, iNOS or an alternatively activated macrophage marker CD206 demonstrated a time-dependent and significant increase in the number of CD206 expressing cells at 9 months of age, when the tumor had formed (**Figure 1D**). Next to assess if tumor-derived nucleic acids can activate the inflammasome in leukocytes in the tumor microenvironment, CD11b+ cells isolated and cultured overnight from MMTV-Wnt+ tumors secreted more IL-1β and expressed higher levels of inflammasome components AIM2 and NLRP3 as compared to CD11b+ cells derived from normal fat pads (**Figures 1E, F**).

### Tumor secretome increases IL-1β production from macrophages

**Figure 2:**
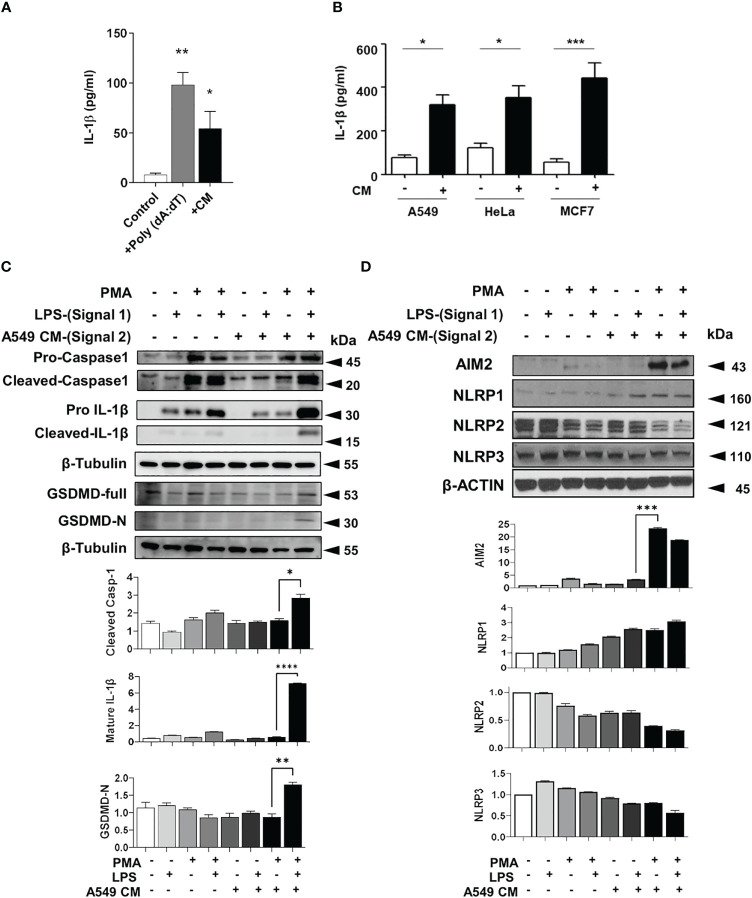
Tumor conditioned media stimulates inflammasomes in macrophages **(A)** IL-1β production in bone marrow derived macrophages primed with LPS and treated with either poly (dA: dT) or TRAMPC2 prostate cancer conditioned media (CM). **(B)** IL-1β production from PMA and LPS treated THP-1 treated with CM from A549, Hela or MCF7 cells (+) or DMEM only for 24 h (-). **(C, D)** Inflammasome components from PMA and LPS treated THP-1 treated with CM from A549 cells. Graph shows the quantification of immunoblots using Image J (fold change vs Actin) *p<0.05; **p<0.01; ***p<0.001; ****p<0.0001. Data represents the mean ± SEM of at 3-5 independent experiments.

To determine if tumors secrete mediators which can enhance the IL-1β and inflammasome activation in macrophages, we collected conditioned media (CM) from TRAMPC2 mouse prostate cancer cells overnight. TRAMPC2 CM induced a high level of IL-1β production in LPS-primed bone marrow derived macrophages (BMDM) (**Figure 2A**). This was confirmed with human monocytic THP-1 cells either resting (monocytic) or differentiated with PMA (macrophage-like) and stimulated with CM from several human cancer cells (**Figure 2B**). Monocytic THP1 cells did not secrete IL-1β in response to LPS or LPS + CM, while macrophage-like THP1 cells secreted more IL-1β basally, when stimulated with LPS and significantly more when stimulated with both LPS+CM (**Figure S2A**). In addition, A549 tumor CM significantly enhanced the cleavage and maturation of inflammasome components caspase-1 and IL-1β and upregulated AIM2 (**Figures 2C, D**). Surprisingly, despite the presence of cytosolic DNA or exogenous DNA transfection, epithelial cancer cells do not produce appreciable amounts of IL-1β compared to myeloid THP-1 cells, implying that the IL-1β in the tumor microenvironment does not originate from the tumor cells themselves (**Figure S2B**).

### Silencing or inhibiting AIM2 or NLRP3 in macrophages reverses IL1β production induced by tumor CM

**Figure 3:**
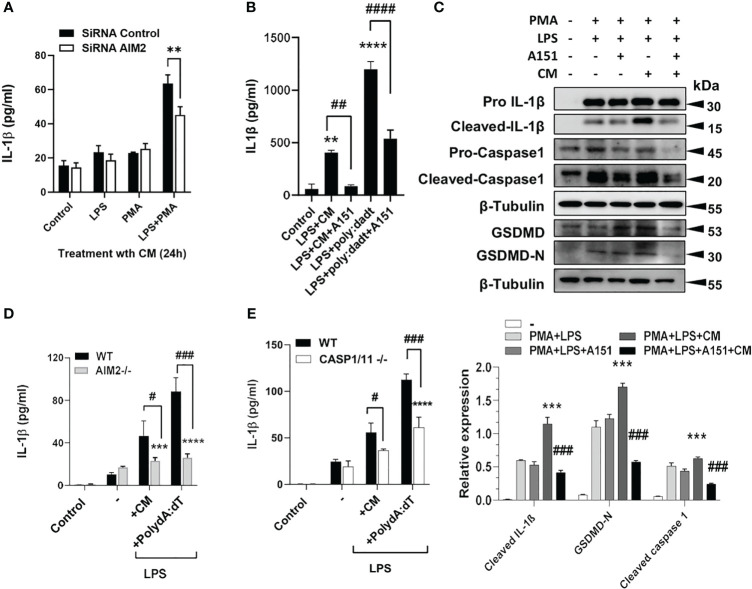
Silencing or inhibiting AIM2 in macrophages reverses IL1β production induced by tumor CM. **(A)** IL-1β levels from control, LPS or PMA Primed THP-1 cells transfected with AIM2 siRNAs or scrambled siRNAs (siRNA Control) and treated with A549 CM. **(B, C)** IL-1β levels and inflammasome activation in LPS and PMA primed THP-1 cells pre-treated with A151 for 1h prior to treatment with A549 CM. Graph shows the quantification of immunoblots using Image J (fold change vs Actin) **(D,E)** IL-1β levels in LPS-primed WT and AIM2-/- or Caspase1/11-/- BMDM treated with mouse TRAMP-C2 CM or Poly(dA:dT). Data represents mean ± SEM of at least 3 independent experiments. **p<0.01; ***p<0.001, **** p<0.0001. # p<0.05; ##p<0.01; ###p<0.001; ####p<0.0001.

Next, AIM2 was either silenced with siRNA ((**Figure 3A**) or treated with A151, an inhibitor of AIM2 and TLR9 (**Figure 3B**) which resulted in the inhibition of IL-1β and inflammatory components after A549 CM in human THP1 cells, with quantified densitometry (**Figure 3C**). AIM2-/- and caspase 1-/- BMDM, treated with LPS and either dsDNA or tumor CM, similarly produced significantly less IL-1β compared to WT BMDM (**Figures 3D, E**). This shows that macrophage AIM2 is important for IL-1β secretion in response to tumor CM. However, as Caspase-1 plays a role here, we examined if other inflammasomes could also be involved. Inhibition of NLRP3 with MCC950 partially inhibited the release and cleavage of IL-1β and induced by LPS+ATP as well as A549 CM, indicating that the NLRP3 inflammasome activation is partially responsible (**Figure S3**).

### AIM2-dependent IL1β production stimulates tumor cell proliferation

**Figure 4:**
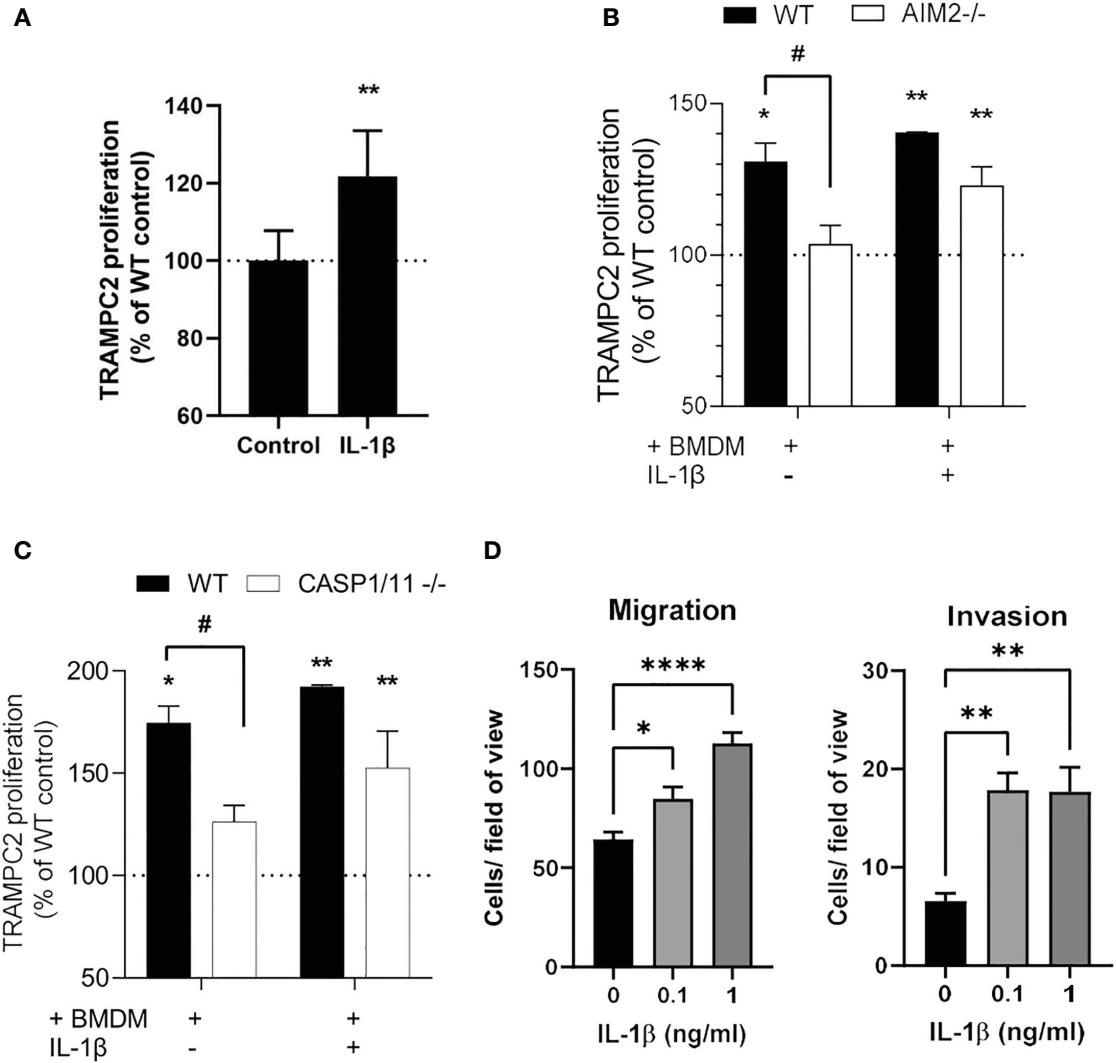
IL1β production from macrophages induces TRAMPC2 proliferation and migration/invasion **(A-C)** Proliferation rates of TRAMPC2 cells co-cultured with C57BL6, AIM2 -/- or Caspase 1/11 -/- bone marrow derived macrophages for 24h, with and without 0.25 ng/ml mouse IL-1β. *p<0.05, ** p<0.01 vs WT control; # p<0.05 vs WT+BMDM **(D)** Migration and invasion of TRAMPC2 cells cultured with IL-1β. Data are from n=3 mice *p<0.05; **p<0.01; ****p<0.0001.

Exogenous IL-1β stimulated proliferation of TRAMPC2 cancer cells, and addition of exogenous IL-1β stimulated proliferation of TRAMPC2 cancer cells similarly in the presence of WT and AIM2-/- or Caspase1/11-/- macrophages (**Figure 4A**). However, coculture of TRAMPC2 cancer cells with BMDM increased their proliferation which was AIM2 and Caspase 1/11 dependent as this was not observed with AIM2-/- or Caspase1/11-/- BMDM (**Figures 4B, C**). In addition, IL-1β enhanced the migration and invasion of TRAMPC2 cancer cells (**Figure 4D**) which implies a pro-tumorigenic role for IL-1β.

### Cytosolic DNA and DNA/RNA hybrids are increased in tumors and colocalize with AIM2

**Figure 5:**
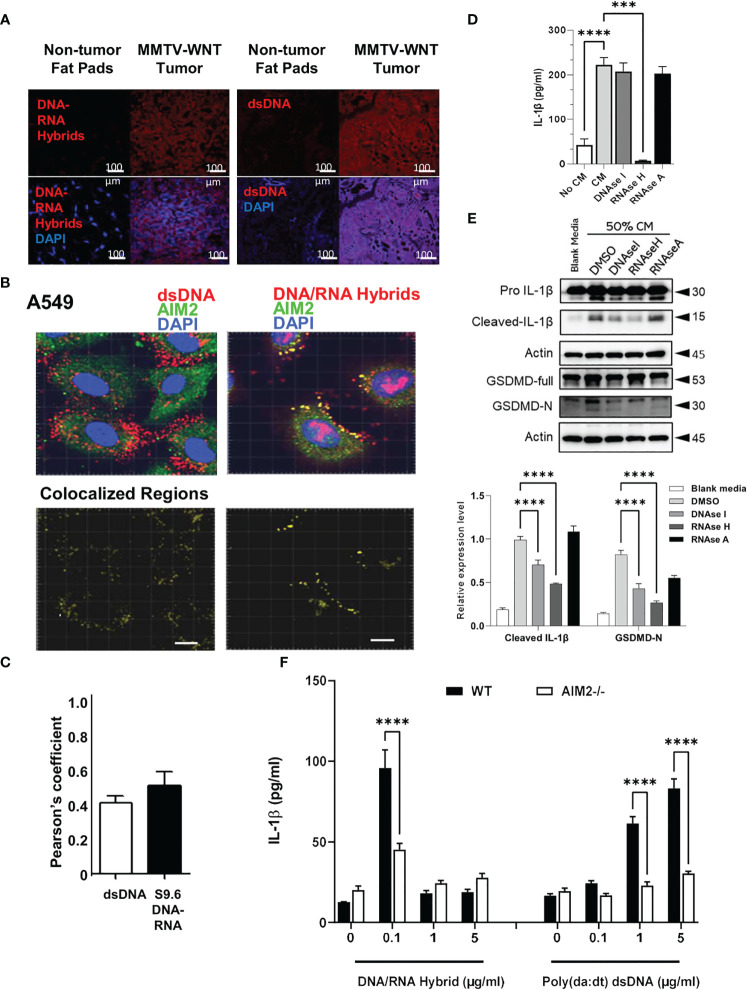
Tumor derived DNA-RNA hybrids activate AIM2 inflammasome. **(A)** MMTV Tissues and non-cancerous fat pads from mice were co-labelled for dsDNA or DNA/RNA hybrids (red) in the presence of DAPI (blue). **(B, C)** A549 cancer cells were co-labelled for AIM2 (green) and dsDNA (red) or -DNA-RNA hybrids (red) in presence of DAPI (blue). Co-localized regions are shown in yellow. Bar=20µm. Pearson’s correlation of colocalization between AIM2 and either dsDNA or S9.6 DNA-RNA hybrids is shown. **(D)** A549 CM was treated with DMSO, sDNAase I (10 μg/ml), RNAase H (10U/ml) or RNAase A (10 μg/ml) for 2h and added to primed and differentiated THP-1 cells for 24h. Cell supernatants were collected and IL-1β levels analyzed using ELISA. **(E)** Cells were treated as above and inflammasome components (IL-1β and Gasdermin-D) and activation (cleaved, GasderminD-N) were analyzed using western blotting. Graph shows the quantification of immunoblots using Image J (fold change vs Actin) **(F)** AIM2-/- and WT BMDMs were treated with LPS (1 μg/ml) for 6h and increasing concentrations of DNA-RNA hybrids or Poly (dA: dT). Oligonucleotides were transfected with lyovec transfection reagent for the indicated time according to the manufacturer’s recommendations. Cell supernatants were collected 24 h post- LPS priming, and IL-1β levels were quantified using ELISA. Data represents the mean ± SEM of at least 3 independent experiments. ***p<0.001; ****p<0.0001.

The presence of dsDNA and DNA-RNA hybrids has been reported in cancer tissues and cell lines (3, 17–19). We hypothesized that these cytosolic nucleic acids may act as intracellular ligands to stimulate AIM2 inflammasome activation. Therefore, we next determined if cytosolic DNA or DNA-RNA hybrids were present in the spontaneous mammary tumors in MMTV-Wnt+mice by staining MMTV-Wnt+ tumors and non-tumor fat pads from MMTV-Wnt- for both DNA-RNA hybrids and dsDNA using immunofluorescence staining. Both cytosolic dsDNA and DNA-RNA hybrids are constitutively present in tumor tissues and are not found in non-tumor mammary fat pads (**Figure 5A**). In addition, AIM2 colocalized with cytosolic DNA and DNA-RNA hybrids in A549 cells (**Figures 5B, C**) as well as other tested cell lines (U937, THP-1, Hela, and HCT-116, **Figure S4**).

To determine which nucleic acid species in the tumor CM could be responsible for inflammasome activation, DNAase, RNAase H, and RNAase A which degrade dsDNA, DNA-RNA hybrids, and dsRNA, respectively, was added to A549 CM prior to addition to THP-1 cells. Tumor CM stimulated the production of IL-1β which was significantly inhibited with RNAase H, and not RNAase A. Interestingly, only a slight reduction was observed with DNAase treatment (**Figure 5D**). Furthermore, treatment with RNAase H significantly reduced the maturation of IL-1β, and inhibited gasdermin D-N expression (**Figure 5E**). Treatment with DNAase, to a smaller extent, reduced the maturation of IL-1β, but did not inhibit the IL1β release, suggesting that DNA-RNA hybrids present in tumor CM, could be responsible for the IL-1β secretion and inflammasome activation in THP1 macrophages, rather than dsDNA.

Finally, to validate that the AIM2 inflammasome can sense dsDNA and DNA-RNA hybrids, BMDM from WT and AIM2-/- mice were generated and transfected with different concentrations of DNA-RNA hybrids or dsDNA poly (dA: dT). The lowest concentration of DNA-RNA hybrid transfection (0.1μg/ml) increased IL-1β secretion which was AIM2 dependent. Interestingly, transfection of DNA-RNA hybrids at higher concentrations (1µg/ml and 5 µg/ml) did not increase IL-1β secretion compared to the transfected control. (**Figure 5F**). However, only higher concentrations of poly (dA: dT) stimulated IL-1β production. This data suggests that AIM2 can respond to both DNA-RNA hybrids and dsDNA, but is more sensitive to DNA-RNA hybrids as compared to dsDNA

In summary, this study shows that macrophage AIM2 is activated by nucleic acids secreted from the cancer cells, which leads to inflammasome activation, IL-1β cleavage and release from macrophages. IL-1β and coculture of tumor cells with macrophages enhances the proliferation of tumor cells which is AIM2 and caspase 1/11 dependent (Gigure 6). AIM2 is more sensitive to stimulation with DNA-RNA hybrids compared to dsDNA, suggesting that both dsDNA and DNA-RNA hybrids can activate AIM2 inflammasome in tumor associated macrophages in the tumor microenvironment.

## Discussion

Understanding the tumor microenvironment and the intrinsic relationship between tumor cells and immune cells is essential for developing potential therapy options. The critical role of inflammation in the tumor microenvironment is beginning to be appreciated, with many tumor types showing an inflammatory signature (20). Although cancer inflammation is described as a double-edged sword (21), it is without question a key mediator of cancer development with both tumor-promoting and inhibiting properties. Inflammasomes are multimeric protein complexes that are key drivers of inflammation and can recognize DNA to trigger innate immune responses. The AIM2 inflammasome is a DNA sensor that has been reported to be activated by dsDNA (22). In particular, the AIM2 inflammasome has been reported to be an important tumor regulator with inflammasome-dependent and independent roles in cancer but its exact role remains unclear (6, 23–25). The complex role of AIM2 in the tumor microenvironment may be more complicated through its inhibition of several signaling pathways including AKT (16) as well as STING-type 1 interferon signaling (7b) Most importantly, how AIM2 gets activated in cancer remains unknown.

A strength of the paper is the use of the MMTV-WNT mouse model as we wished primarily to look at spontaneous cancer growth. The MMTV-WNT mouse develops tumors at ~6-9 months of age so it was possible to determine if the numbers of infiltrating macrophages increases with time pre-tumor. *In vitro*, other tumor cell lines were used, such as human Hela (cervical cancer), human HCT116 (colon cancer) and human A549 (lung cancer) as well as mouse TRAMPC2 (prostate cancer). This demonstrates that all cancer cell lines could stimulate AIM2 inflammasome activation in macrophages exposed to the conditioned media from these cell lines. Thus our observations are not tumor type specific, but we believe a reflection of cancer cells in general.

The role of IL-1β in cancer has both positive and negative functions and may be dependent on the cancer type and stage. Our study has shown that IL-1β enhances proliferation of prostate cancer cells at physiological concentrations. This is in line with studies showing that host IL-1β is required for *in vivo* angiogenesis and invasiveness of melanoma, prostate and breast tumor cells through the production of VEGF and cytokines by macrophages(26). Similarly, IL1 was shown to be produced by tumor infiltrating myeloid cells in the tumor microenvironment and neutralizing IL1 receptor inhibits breast cancer progression *in vivo* in a humanized mouse model(27), indicating the importance of paracrine IL-1β in tumor growth and progression. In other studies, monocyte-derived IL-1β inhibited prostate cancer proliferation and induced apoptosis(28). However, interestingly, IL-1β inhibits differentiation and metastasis of metastasis initiating cells and among patients with lymph node-positive breast cancer, high primary tumor IL-1β expression is associated with better overall survival and distant metastasis-free survival (29 *)*.

The other players in the inflammasome pathway such as Gasdermin-D are is also involved in the macrophage inflammasome-dependent proliferation of cancer where lung metastasis in Gasdermin D -/- mice was reduced (30). Similar to our study, macrophage gasdermin-D plays a role in the tumor microenvironment. In addition, Caspase-1 and Gasdermin-D deficient macrophages enhance the activation of cGAS-STING dependent type-1 interferon pathway. However, this was reported to be pytoptosis, IL1 and IL-18 independent and through K+ depletion and suppression of cGAS activation (31). NLRP3 also has been reported in a multitude of studies to be involved in tumor growth and development both as an oncogene and as a tumor suppressor gene. (32). The spatial and temporal activation and expression of AIM2 and NLRP3 in varying tumor types may explain the conflicting reports but more studies are needed to fully understand the importance of the inflammasomes and its components in cancer.

Although implicated in different types of cancer, the trigger for AIM2 in these cancers remains unknown. Our results show that DNA-RNA hybrids may be the trigger for AIM2 inflammasome in cancers. Firstly, macrophages exhibited a significant decrease in IL-1β release when treated with DNA-RNA hybrid-depleted tumor conditioned media. This effect is not seen using dsDNA-depleted tumor conditioned media. Secondly, AIM2 -/- macrophages have impaired levels of IL-1β following transfection with DNA-RNA hybrids. Thirdly, immunostaining revealed that AIM2 colocalizes with cytosolic DNA-RNA hybrids to a larger extent than dsDNA in various cells tested. Taken together, our results indicate AIM2 indeed interacts with DNA-RNA hybrids. To the best of our knowledge, DNA-RNA hybrids have not been previously linked to AIM2 inflammasome in cancer. However, AIM2 was reported to be activated by RNA viruses such as influenza viruses (33, 34), Chikungunya virus and West Nile virus (35). Given that DNA-RNA hybrids are generated as intermediates during replication of the various viruses within infected cells (36, 37), RNA viruses may activate AIM2 via direct sensing of the DNA-RNA hybrids produced when the viral RNA is reverse transcribed into DNA. However, one report finds that bacterial DNA-RNA hybrids from Enterohemorrhagic Escherichia coli are sensed by NLRP3 inflammasome (38). Our preliminary data indicates that while tumors express high levels of AIM2 and NLRP3, tumor CM increases the expression of AIM2, but not NLRP1, NLRP2 or NLRP3 inflammasomes, suggesting that AIM2 and NLRP3 inflammasomes may play complementary roles in inflammasome activation in response to tumor-derived mediators. It is important to note that our model here reports AIM2 sensing of endogenous self-DNA-RNA hybrids and not foreign bacterial hybrids.

Given the understanding that AIM2 recognizes dsDNA (10, 39), our study is one of the first to describe AIM2 recognition of DNA-RNA hybrids and at a higher sensitivity than dsDNA. It may be possible that AIM2 is only capable of sensing dsDNA more than 80bp in length (40). While the length of dsDNA in the tumor cells we used (A549, Hela, MCF-7) remains elucidated, it is certainly possible that the dsDNA present in these tumor cells is too short to trigger AIM2 activation in this model.

**Figure 6:**
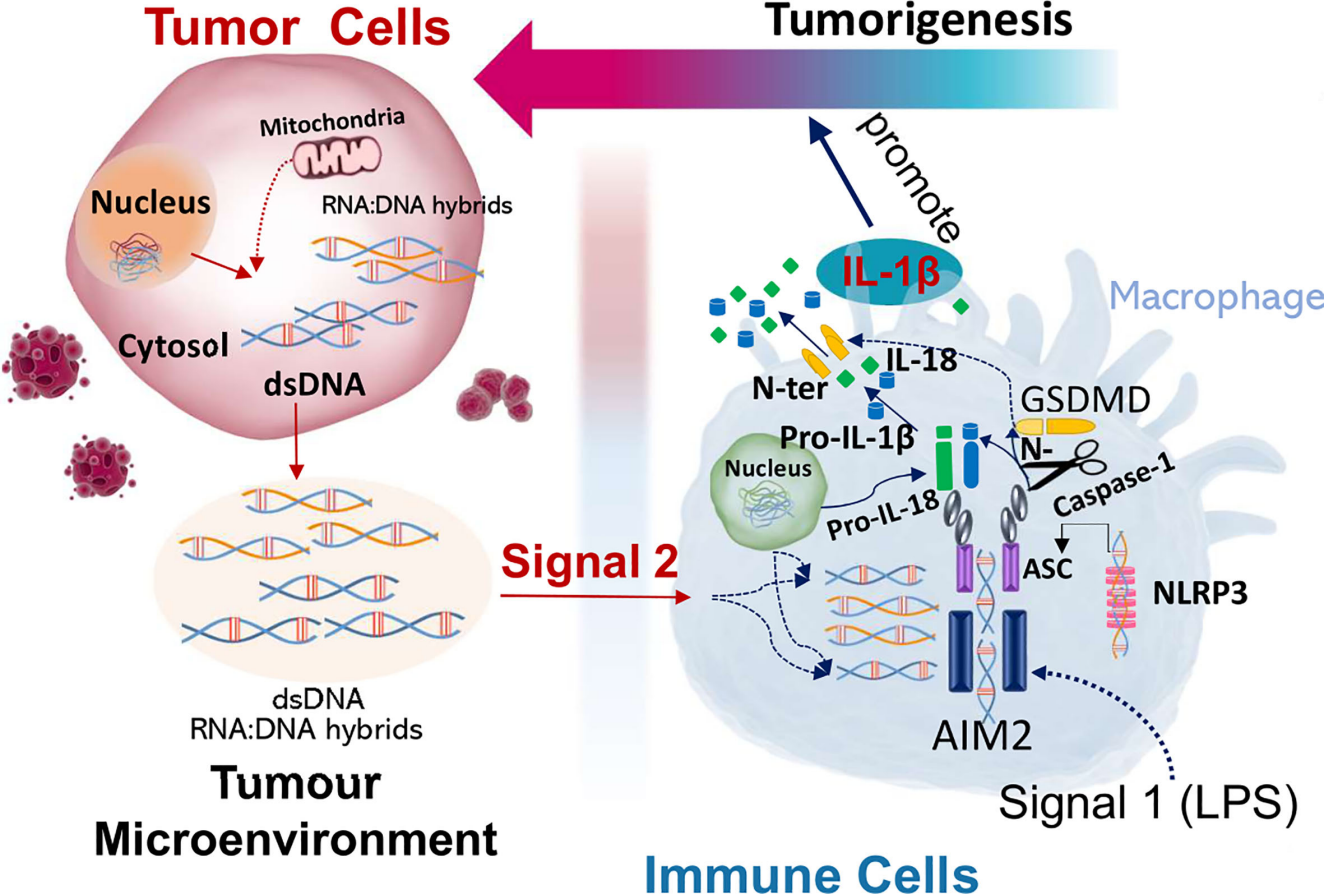
DNA-RNA hybrids and dsDNA in the tumor microenvironment activate and trigger AIM2 induced inflammation in macrophages to produce a pro-tumor response. Cancer cells secrete cytosolic DNA into the tumor microenvironment which is taken up by the macrophages. DNA-RNA hybrids and dsDNA bind and activate AIM2 inflammasome in macrophages, which produce pro-tumorigenic responses.

In summary, we show that tumors express cytosolic DNA and have higher numbers of infiltrating macrophages. Macrophages release IL-1β indicative of inflammasome activation after stimulation with LPS and tumor-CM, which is AIM2 dependent. IL-1β and coculture of tumor cells with macrophages enhances the proliferation of tumor cells which is AIM2 and Casp1/11 dependent. We propose that cancer cells release nucleic acids into the tumor microenvironment, which activates macrophages to produce an acute pro-tumor response (**Figure 6**). Accordingly, understanding paracrine tumor cytosolic DNA and immune cell interactions in cancer pathogenesis is a critical step for therapeutic development. Broader understanding of the mechanisms of nucleic acid interaction with innate immune signaling pathways would provide novel targets for anticancer research and future molecular therapy.

## Data availability statement

The original contributions presented in the study are included in the article/**Supplementary Material**. Further inquiries can be directed to the corresponding author.

## Ethics statement

The animal study was reviewed and approved by National University of Singapore IACUC, (AAALAC-accredited institution).

## Author contributions

ZC, LL: Conception and design. ZC, JC, KaS, KrS, and KK: Investigation. ZC, JC, and LL: Analysis and interpretation of data. ZC: Roles/Writing original draft. JC, SG, and LL: Writing, review & editing. HL, AK, SL: Administrative, technical, or material support. LL, and SG: Study supervision and funding acquisition. All authors contributed to the article and approved the submitted version.
