# Supplementary material for: Macrophage IL-1β contributes to tumorigenesis through paracrine AIM2 inflammasome activation in the tumor microenvironment
Source: Front Immunol. 2023 Jun 28;14:1211730. doi: 10.3389/fimmu.2023.1211730 (PMC10338081; doi:10.3389/fimmu.2023.1211730)
Supplement: Supplementary file 1 [file DataSheet_1.pdf]

Supplementary Materials for

Macrophage IL1β contributes to tumorigenesis through paracrine AIM2 inflammasome activation in the tumor microenvironment

Zhi Huan Chew[1,2,3,4, †], Jianzhou Cui[1,2,3, †], Karishma Sachaphibulkij[1,2,3], Isabelle Tan[1,2,3], Shreya Kar[5,6], Kai Kiat Koh[1,2,3], Kritika Singh[5], Hong Meng Lim[1,2,3], Soo Chin Lee[5,7,8], Alan Prem Kumar[5,6,8], Stephan Gasser[3,9], Lina H. K. Lim[1,2,3,4]

*Corresponding author. Lina H. K. Lim Email:

This file includes:

Figs. S1 to S4

Supplementary figures

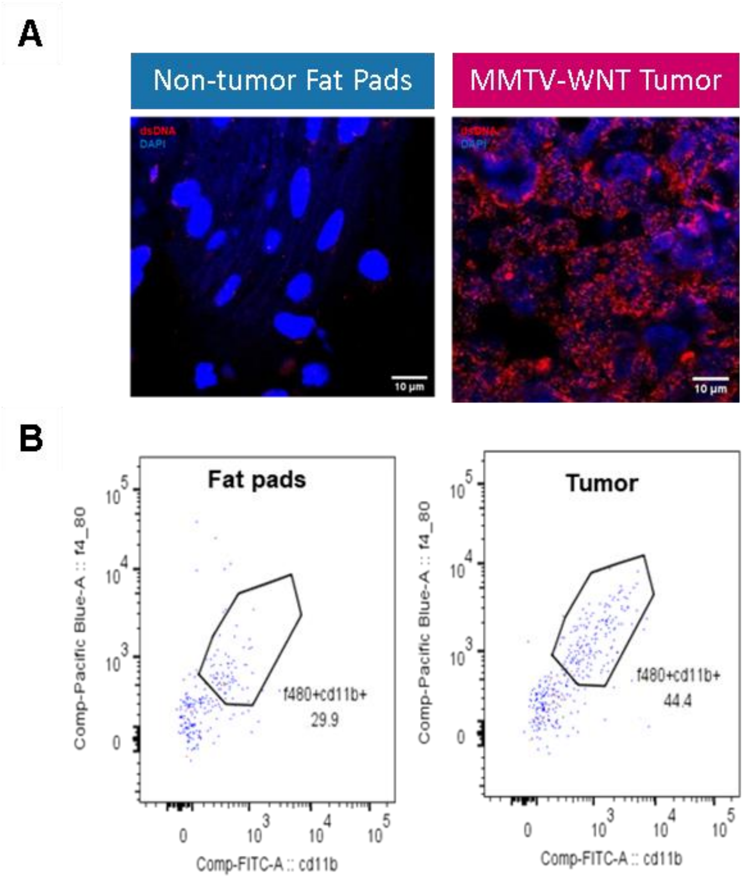

Fig. S1. The difference of dsDNA between MMTV-Wnt+mice and non-tumor fat pads. (A) MMTV Tissues and non-cancerous fat pads from mice were co-labelled for dsDNA (red) in the presence of DAPI (blue). (B) Representative flow cytometric analysis showing the gating strategy to identify Live CD45+F4/80+CD11b+ tumour-associated macrophages (TAM) from tumors of MMTV-Wnt+ mice and normal mouse fat pads from MMTV-Wnt- mice. Data are representatives from n=3-5 mice.

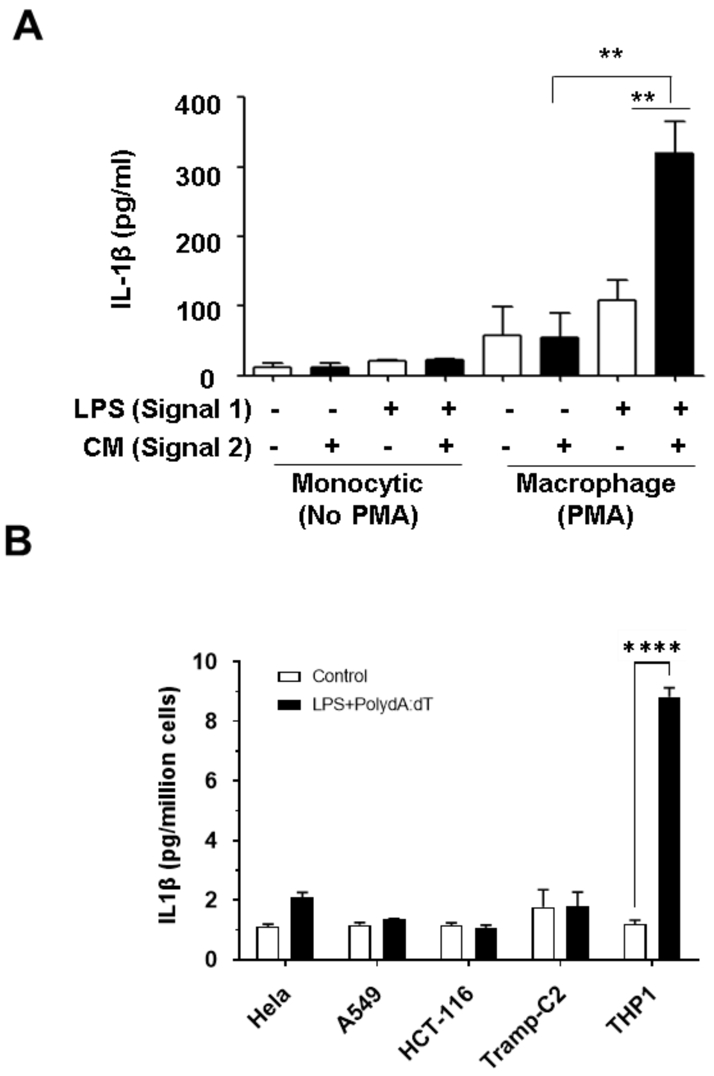

Fig. S2. Inflammasome activation optimization in macrophages and epithelial cells. (A) THP-1 cells were treated with or without PMA (100ng/ml), LPS (1µg/ml) for 24h, followed by A549 conditioned media, or DMEM only for 24h. IL-1β levels in cell supernatants were analyzed using ELISA. (B) IL1β production was measured in various epithelial cancer cells and THP1 cells after treatment with LPS and Poly:datdt. Data represents mean ± SEM of 3-5 independent experiments. **P<0.01, **** p<0.00001

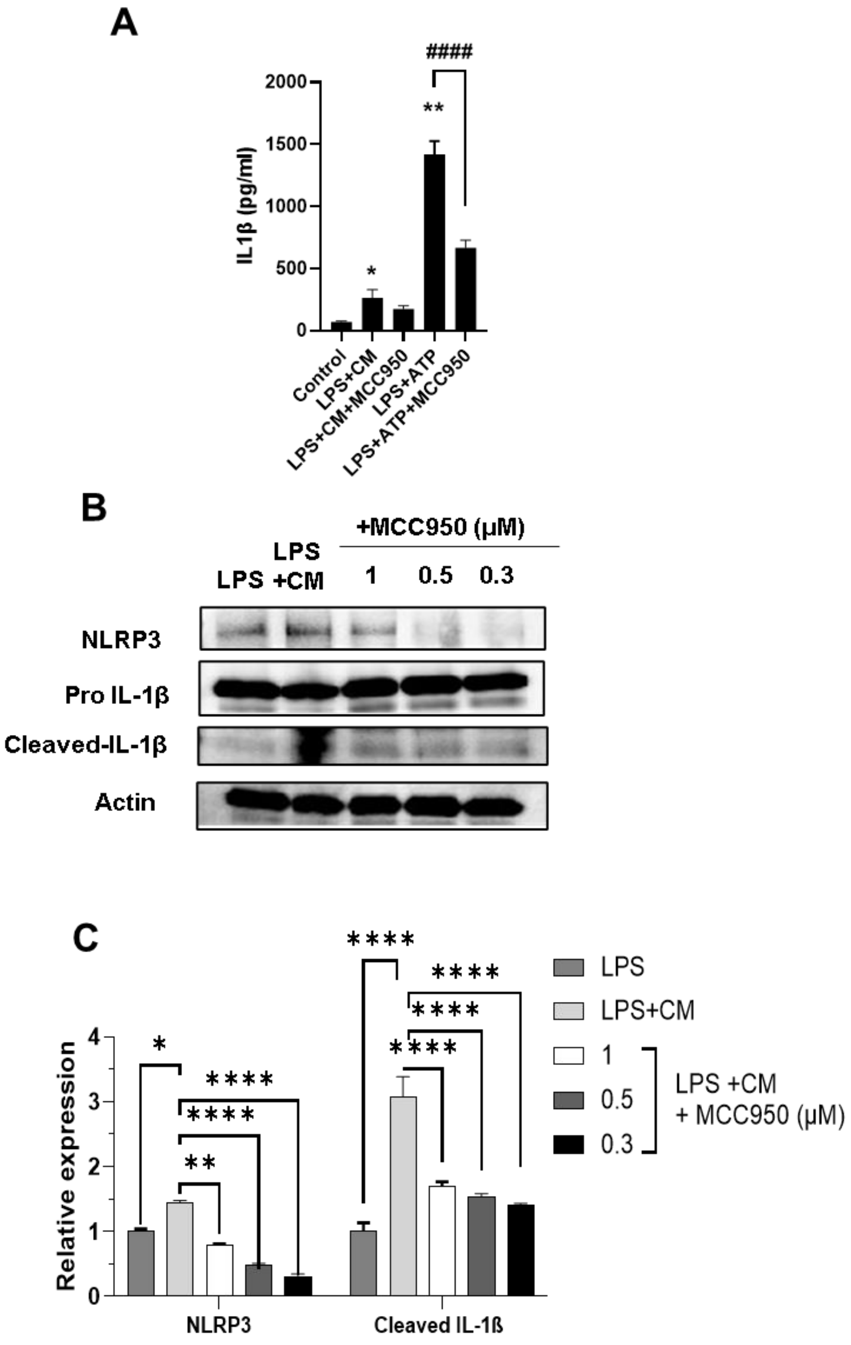

Fig S3. Inhibition of NLRP3 with MCC950 reverses IL1β production induced by tumor CM. (A) IL-1β levels and (B) inflammasome activation in LPS and PMA primed THP-1 cells pre-treated with MCC950 for 1h prior to treatment with A549 CM. (C) Quantification of immunoblots using Image J (fold change vs loading control) * # $p<0.05$; ** ## $p<0.01$; *** ### $p<0.001$ **** #### $p<0.0001$.

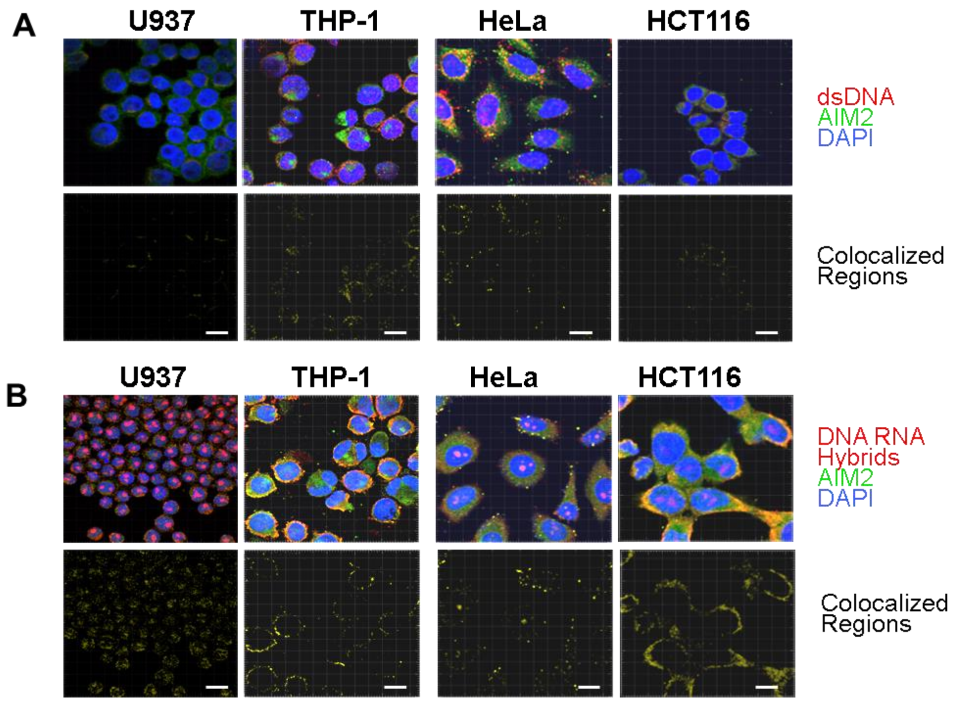

Fig S4. AIM2 colocalizes with dsDNA and DNA-RNA hybrids in various cancer cell lines. U937, THP1, HeLa and HCT116 cancer cells were co-labelled for AIM2 (green) and dsDNA (red) or -DNA-RNA hybrids (red) in presence of DAPI (blue). Co-localized regions are shown in yellow. Bar=20µm.
